# AlGaN/GaN High Electron Mobility Transistors on Semi-Insulating Ammono-GaN Substrates with Regrown Ohmic Contacts

**DOI:** 10.3390/mi9110546

**Published:** 2018-10-25

**Authors:** Wojciech Wojtasiak, Marcin Góralczyk, Daniel Gryglewski, Marcin Zając, Robert Kucharski, Paweł Prystawko, Anna Piotrowska, Marek Ekielski, Eliana Kamińska, Andrzej Taube, Marek Wzorek

**Affiliations:** 1 Institute of Radioelectronics and Multimedia Technology, Warsaw University of Technology, Nowowiejska 15/19, 00-662 Warsaw, Poland; M.Goralczyk@ire.pw.edu.pl (M.G.); dgrygle@ire.pw.edu.pl (D.G.); 2 Ammono Lab, Institute of High Pressure Physics, Polish Academy of Sciences, Sokołowska 29/37, 01-142 Warsaw, Poland; zajac@ammono.com (M.Z.); kucharski@ammono.com (R.K.); 3 TopGaN Ltd., Sokołowska 29/37, 01-142 Warsaw, Poland; pawel.prystawko@unipress.waw.pl; 4 Institute of High Pressure Physics, Polish Academy of Sciences, Sokołowska 29/37, 01-142 Warsaw, Poland; 5 Institute of Electron Technology, Al. Lotników 32/46, 02-668 Warsaw, Poland; ekielski@ite.waw.pl (M.E.); ataube@ite.waw.pl (A.T.); mwzorek@ite.waw.pl (M.W.)

**Keywords:** high electron mobility transistors, high electron mobility transistor (HEMT), AlGaN/GaN, ohmic contact, regrown contact, ammonothermal GaN, power amplifier

## Abstract

AlGaN/GaN high electron mobility transistors on semi-insulating bulk ammonothermal GaN have been investigated. By application of regrown ohmic contacts, the problem with obtaining low resistance ohmic contacts to low-dislocation high electron mobility transistor (HEMT) structures was solved. The maximum output current was about 1 A/mm and contact resistances was in the range of 0.3–0.6 Ω·mm. Good microwave performance was obtained due to the absence of parasitic elements such as high access resistance.

## 1. Introduction

There is a consensus in the nitride community that, although several GaN-based devices have already reached the market, their properties are still inferior with respect to predicted performance [1]. There are still many technological issues to be faced in order to fully exploit the enormous potential of these materials. The main limitations come from the lack of large area native bulk GaN substrates of reasonable cost and quality for homoepiaxial growth; some other issues concern device processing. Here, reliable low resistance homogeneous Ohmic contacts being fundamental building blocks of GaN devices are highly required.

In the quest to push the performance limit of AlGaN/GaN high electron mobility transistors (HEMTs), our work on advanced devices focuses on two main areas: development of epitaxial growth of HEMT structures on low defect density and high quality bulk ammonothermal semiinsulating GaN and fabrication of compatible nonalloyed ohmic contacts with subcontact n${}^{+}$-In${}_{x}$Ga${}_{1-x}$N:Si epilayer regrown by metal organic vapor phase epitaxy (MOVPE).

The AlGaN/GaN HEMT structures for high power applications are usually grown on silicon carbide substrates [2], but recently there has been significant progress in developing high quality GaN substrates with low defect density using hydride vapor phase epitaxy and ammonothermal growth techniques [3,4,5,6,7]. In particular, truly bulk ammonothermal GaN substrates could be used for homoepitaxy of transistor active layers characterized by excellent crystal quality and low surface roughness due to low threading dislocation density at the order of 1 × 10${}^{4}$ cm${}^{-2}$ [8] and negligible bow. This can lead to improved reliability, radiation hardness, high yield and repeatability of the parameters of the final devices [9,10,11,12].

In addition, due to the reliability and performance issues, the problem of self-heating and heat dissipation inside the epitaxial structure of GaN-based HEMTs is especially important for high power devices. While the thermal conductivity is higher for 4H-SiC than for bulk GaN, the heat flow inside typical GaN-based HEMT on the SiC substrate is significantly limited because of the presence of nucleation layers (e.g., AlN) between epilayers and SiC substrate. This effect is commonly called thermal boundary resistance (TBR) [13]. Dislocations at the interfaces have a large share in the TBR [14]. In the case of AlGaN/GaN structure grown on the bulk gallium nitride, the thermal resistance of GaN-based HEMT is only determined by the thermal conductivity of bulk GaN, and temperature rise under operating conditions can be comparable to that in devices on SiC substrates [15].

While preliminary results on some aspects of device technology were reported [16], AlGaN/GaN HEMTs on ammonothermal GaN substrates with satisfying DC and RF parameters have not yet been published. Moreover, difficulties in obtaining low resistivity ohmic contacts to HEMT structures made on a substrate with a lower dislocation density were reported [17]. In this work, we present AlGaN/GaN high electron mobility transistors on semi-insulating bulk ammonothermal GaN substrates with nonalloyed regrown ohmic contacts. By using metal organic vapor phase epitaxy (MOVPE)-regrown highly-doped n${}^{+}$-In${}_{x}$Ga${}_{1-x}$N:Si layers, low resistivity ohmic contacts (R${}_{c}$∼0.3–0.6 Ω·mm) and high output current (1 A/mm) along with satisfying RF parameters are obtained.

## 2. Experimental Details

**Figure 1:**
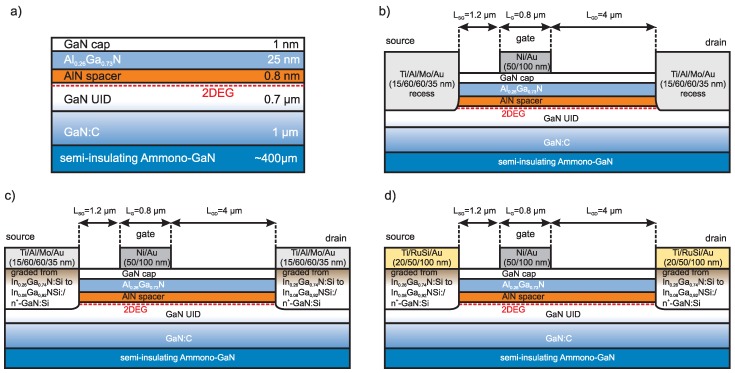
Cross-sectional schematics of the AlGaN/GaN-on-Ammono GaN high electron mobility transistors (HEMTs) under study: semiconductor device structure (**a**) and HEMT layouts with recessed Ti/Al/Mo/Au ohmic contact; (**b**) with subcontact n${}^{+}$-In${}_{x}$Ga${}_{1-x}$N:Si regrown epilayer and Ti/Al/Mo/Au ohmic contact (**c**), and with subcontact n${}^{+}$-In${}_{x}$Ga${}_{1-x}$N:Si regrown epilayer and Ti/RuSi/Au ohmic contact (**d**).

HEMT structures used in this study (see Figure 1a) were grown on a 1-inch c-plane, ∼400 μm thick, semi-insulating ammonothermal bulk GaN (SI Ammono-GaN) substrates. The resistivity of semi-insulating Ammono-GaN is typically no less than 10${}^{9}$ Ω·cm in paralel direction to the c-axis, as measured by frequency domain capacitive technique [18,19], and over 1 × 10${}^{6}$ Ω·cm (above the measurement method range) in the perpendicular direction to c-axis, as determined by microwave methods [19,20].

High resistivity of SI Ammono-GaN substrates are obtained by compensation of residual oxygen, incorporated during ammonothermal growth, by deep acceptors i.e., transition metal ions or by Mg shallow acceptors. It is worth to noting that a low level of impurities (∼2 × 10${}^{18}$ cm${}^{-3}$) contributes to the high value of room temperature thermal conductivity of semi-insulating Ammono-GaN (κ∼230 W/mK) [21]. The HEMT structure was grown by MOVPE. It consists of 1 nm GaN-cap, 25 nm Al${}_{0.26}$Ga${}_{0.73}$N barrier layer, 0.8 nm AlN spacer, 0.7 μm unintentionally doped (UID) GaN and 1 μm GaN:C highly resistive buffer. As shown in Figure 1a a dotted line indicates the position of two-dimensional electron gas (2DEG) formed in the quantum well at AlN spacer/UID GaN interface.

**Figure 2:**
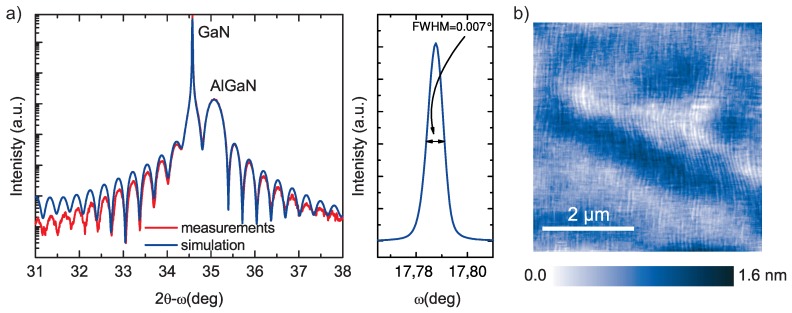
(**a**) 2θ-ω high resolution X-ray diffraction (XRD) scan and XRD rocking curve along with (**b**) AFM image of the surface of AlGaN/GaN HEMT structure on SI Ammono-GaN substrate.

The high resolution 2θ-ω and rocking curve X-ray diffraction (XRD) scans of epilayers (Figure 2a) prove the excellent crystal quality of epilayers grown on SI Ammono-GaN with FWHM = 0.007°. Atomic force microscopy (AFM) scans (5 µm × 5 µm) of the top of AlGaN/GaN HEMT structure shows an atomically smooth surface with a root mean square roughness of about 0.12–0.14 nm (Figure 2b). Electrical parameters of 2DEG were obtained by Hall effect and C-V measurements. Sheet resistivity (R${}_{sh}$), sheet carrier concentration (n${}_{s}$) and Hall mobility (μ) were 315 Ω/☐, 1.64 × 10${}^{13}$ cm${}^{-2}$ and 1210 cm${}^{2}$/Vs, respectively.

The first step of HEMT processing was the deposition on the semiconductor device structure a double-layer SiO${}_{x}$ (200 nm)/AlN (35 nm) mask and its patterning for the selective recess etching of AlGaN/GaN followed by selective MOVPE regrowth of n${}^{+}$-In${}_{x}$Ga${}_{1-x}$N:Si/n${}^{+}$-GaN:Si subcontact regions of source and drain. The SiO${}_{x}$ film was deposited using plasma-enhanced chemical vapor deposition (PECVD) and AlN layer was grown by MOVPE. Mask patterning was performed by BCl${}_{3}$/Ar and CHF${}_{3}$/CF${}_{4}$ plasma etching. The depth of recess etching was 20 nm below the AlGaN layer. The doping and thickness of subcontact regrown region was as follows: n${}^{+}$-GaN:Si (Si: 1.7 × 10${}^{19}$ cm${}^{-3}$–40 nm, 5 × 10${}^{19}$ cm${}^{-3}$–7 nm) and graded (*x* from 8% to 26% at the top) n${}^{+}$-In${}_{x}$Ga${}_{1-x}$N:Si (Si: 5 × 10${}^{19}$ cm${}^{-3}$–10 nm). Schematic cross-section is presented in Figure 1c,d. To lower the surface barrier, doping of the first n${}^{+}$-GaN:Si layer was kept below Mott concentration, while the next 7 nm n${}^{+}$-GaN:Si layer was doped to the higher level of Si. For further lowering the surface barrier, n${}^{+}$-In${}_{x}$Ga${}_{1-x}$N:Si graded layer was doped to 5 × 10${}^{19}$ cm${}^{-3}$ of Si and indium composition was chosen in such a way that with 26% of In, the Fermi level is pinned to the conduction band.

**Figure 3:**
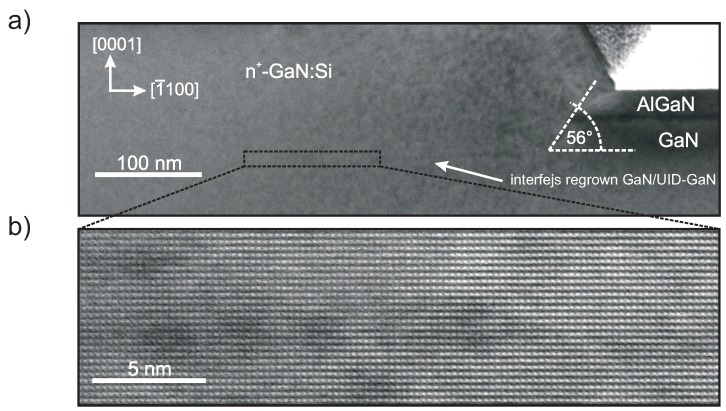
(**a**) TEM image of n${}^{+}$-In${}_{x}$Ga${}_{1-x}$N:Si/n${}^{+}$-GaN:Si subcontact region and (**b**) HR-TEM image of n${}^{+}$-GaN:Si/UID GaN interface.

The AlN/SiO${}_{2}$ mask was removed by soaking in hydrofluoric acid solution while regrown nitride films remained in contact regions. Figure 3 shows a cross-sectional transmission electron microscope (TEM) images of regrown GaN on top of a low-dislocation GaN homoepitaxial epilayer. High resolution imaging (HR-TEM) at Figure 3b reveals a smooth, dislocation-free n${}^{+}$-GaN/UID GaN interface, the key attribute of GaN on GaN technology. The etched sidewalls are at a 56${}^{\circ }$ angle to the c-plane (0001). The sidewall angle is close to the optimal 62${}^{\circ }$ angle at which the density of dangling bonds on the etched surface is similar to a c-plane surface [22].

Next, the ohmic contact metallization was sputter-deposited and annealed. To compare the properties of alloyed and ohmic contacts, we studied the characteristics of conventional recessed Ti/Al/Mo/Au (15/60/60/35 nm) metallization annealed at 850 °C for 30 s in a nitrogen flow (Figure 1b) with contacts with subcontact n${}^{+}$-In${}_{x}$Ga${}_{1-x}$N:Si regrown epilayer metallized using conventional Ti/Al/Mo/Au (15/60/60/35 nm) (Figure 1c) or thermally stable Ti/RuSi/Au (20/50/100 nm) metallizations(Figure 1d).

In the following, the isolation of adjacent devices was done by using two-step Al${}^{+}$ ion implantation [23]. The implant consisted of Al ions at (1st step) energy of 800 keV, and dose 1.5 × 10${}^{13}$ cm${}^{-2}$ and (2nd step) at energy 300 keV, and dose 1 × 10${}^{13}$ cm${}^{-2}$. The sheet resistivity of as-implanted isolation was 10${}^{11}$ Ω/☐. With this technique, a sufficiently high vacancy density was obtained in the surface region down to 0.7 μm. To prevent the active regions from becoming implanted, a 3 μm thick photoresist mask was applied. Then, rectangular gate electrodes were an electron-beam deposited Ni/Au (50/100 nm) bilayer. Finally, the devices were passivated by 100 nm SiN${}_{x}$ layer deposited by plasma-enhanced chemical vapor deposition. Finally, windows for contact pads were opened and pads were thickened by Au evaporation. The cross-section schematic of fabricated devices are shown in Figure 1b–d. The gate length (L${}_{G}$) was 0.8 μm and gate width was 2 × 200 μm for two-finger devices. The source-gate (L${}_{SG}$) and gate-drain distance (L${}_{GD}$) were 1.2 μm and 4 μm, respectively.

## 3. Results and Discussion

### 3.1. Electrical Characterisation of Ohmic Contacts with Subcontact n${}^{+}$-In${}_{x}$Ga${}_{1-x}$N:Si Regrown Epilayer to AlGaN/GaN Heterostrucutres on Semi-Insulating Ammono-GaN Substrates

As already mentioned in the Introduction, fabrication of low resistivity ohmic contacts to higher quality HEMT structures appears to be a difficult task. According to numerous studies of Ti/Al-based alloyed contacts to AlGaN/GaN 2DEG, the mechanism of ohmic contact formation is related to spiking through dislocations. Thus, the likely explanation of difficulties is the limited availability of dislocations in reduced defect density HEMTs [17,24,25].

**Figure 4:**
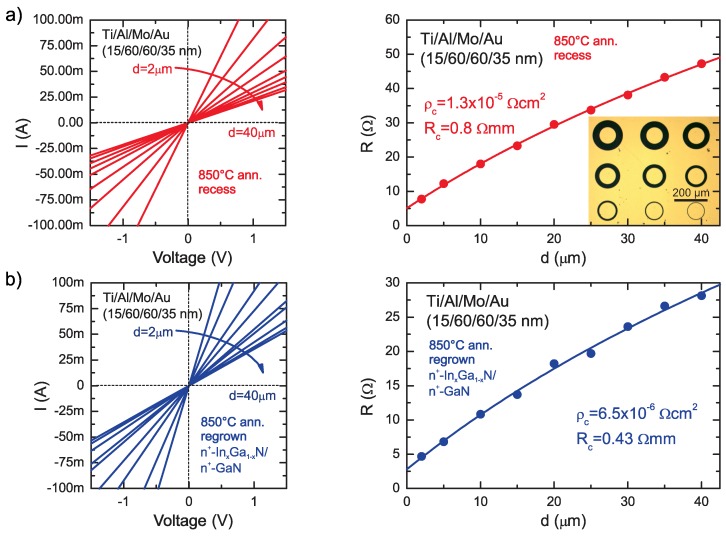
Four-point probe I-V plot of circular transmission line method (CTLM) patterns (image in inset) and measured resistance R vs. CTLM contact spacing, (the solid line is the result of fitting to experimental data) for (**a**) recessed and (**b**) Ti/Al/Mo/Au ohmic contacts with subcontact n${}^{+}$-In${}_{x}$Ga${}_{1-x}$N:Si regrown epilayer annealed at 850 °C.

One of the approaches to overcome this problem is to form a recess below 2DEG and form alloyed Ti/Al-based contact [26,27]. This method was reported successful for HEMTs with dislocation density above 10${}^{6}$ cm${}^{-2}$ i.e., epistructures grown on SiC, Si or even HVPE (hydride vapour phase epitaxy) GaN substrates. In our case, the ohmics resistance of recessed alloyed Ti/Al/Mo/Au contacts to HEMT structures on SI Ammono-GaN are in the range of 0.8–1.1 Ω·mm. As a example, as shown in Figure 4a, the contact resistance and resistivity extracted from circular transmission line method (CTLM) measurements [28] for recessed Ti/Al/Mo/Au ohmic contacts were R${}_{C}$ = 0.8 Ω·mm and $\rho_{c}$ = 1.3 × 10${}^{-5}$ Ω·cm${}^{2}$.

Alloyed Ti/Al/Mo/Au ohmic makes contact with subcontact n${}^{+}$-In${}_{x}$Ga${}_{1-x}$N:Si regrown epilayer show resistances from the range of 0.3–0.6 Ω·mm. For the HEMT structure used in this work, the contact resistance and resistivity were R${}_{C}$ = 0.43 Ω·mm and $\rho_{c}$ = 6.4 × 10${}^{-6}$ Ω·cm${}^{2},$ respectively, as extracted from CTLM measurements(Figure 4b). It is worth mentioning that measured contact resistance presents an upper limit of actual contact resistance as the measured value also includes the contribution of the n${}^{+}$-GaN access region and regrown n${}^{+}$-GaN-2DEG interface [29].

**Figure 5:**
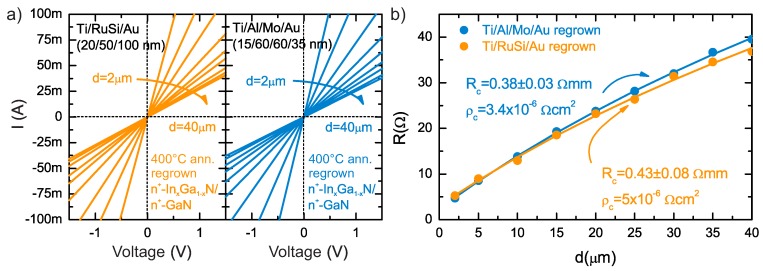
Four-point probe I-V plot of CTLM patterns (**a**) and measured resistance R vs. CTLM contact spacing (**b**) (the solid line is the result of fitting to experimental data) of Ti/Al/Mo/Au and Ti/RuSi/Au ohmic contacts with subcontact n${}^{+}$-In${}_{x}$Ga${}_{1-x}$N:Si regrown epilayer annealed at 400 °C.

The non-alloyed ohmic contacts with Ti/Al/Mo/Au and Ti/RuSi/Au metallizations with subcontact n${}^{+}$-In${}_{x}$Ga${}_{1-x}$N:Si regrown epilayer were mildly annealed at 400 °C for 1 min, in N${}_{2}$ for promoting the adhesion. The comparison of current-characteristics and determination of contacts parameters using CTLM method is depicted in Figure 5. The contacts’ resistance and reactivities obtained from the CTLM method were R${}_{C}$ = 0.38 ± 0.3 ($\rho_{c}$ = 3.4 × 10${}^{-6}$ Ω·cm${}^{2}$) Ω·mm and R${}_{C}$ = 0.43 ± 0.8 Ω·mm ($\rho_{c}$ = 5 × 10${}^{-6}$ Ω·cm${}^{2}$) for Ti/Al/Mo/Au and Ti/RuSi/Au contacts, respectively.

The use of regrown highly-doped In${}_{x}$Ga${}_{1-x}$N/GaN:Si makes it possible to create non-alloyed ohmic contacts to AlGaN/GaN heterostructures without high temperature annealing. Moreover, it allows for using thermally stable metal schemes, which allows for fabricating devices designed for high temperature applications. Sputter-deposited RuSi layers owing to amorphous microstructure [30,31] and high melting point, large work function and low resistivity are the material of choice for a diffusion barrier layer in metallization schemes They have already been proven reliable and thermally stable in GaN-based devices [32].

### 3.2. Electrical Characterization of AlGaN/GaN HEMTs on Semi-Insulating Ammono-GaN Substrates with Ohmic Contacts with Subcontact n${}^{+}$-In${}_{x}$Ga${}_{1-x}$N:Si Regrown Epilayer

**Figure 6:**
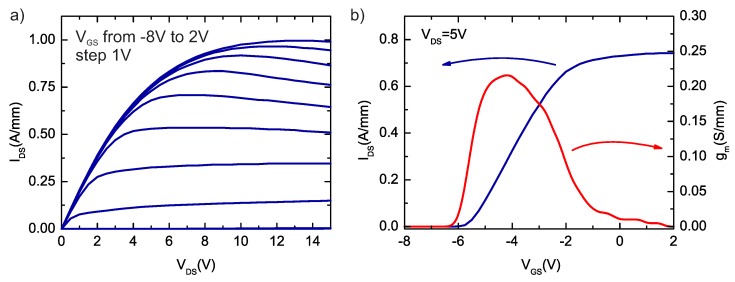
(**a**) output and (**b**) transfer characteristics of fabricated AlGaN/GaN HEMT on SI Ammono-GaN substrate with Ti/Al/Mo/Au ohmic contacts with subcontact n${}^{+}$-In${}_{x}$Ga${}_{1-x}$N:Si regrown epilayer, annealed at 850 °C.

The output and transfer characteristics of the devices (with Ti/Al/Mo/Au ohmic contacts with subcontact n${}^{+}$-In${}_{x}$Ga${}_{1-x}$N:Si regrown epilayer, annealed at 850 °C) are depicted in Figure 6a,b. The maximum drain current density for V${}_{GS}$ = 2 V is about 1 A/mm. Extracted on-state resistance R${}_{on}$ was 4.4 Ω·mm. The kink effect on the output characteristics is not observed. This effect is usually attributed to slow traps located in GaN buffer layer under gate region [33], which was described in previous reports on AlGaN/GaN HEMTs on ammonothermal bulk GaN [17]. The negative slope in the output characteristics for higher V${}_{DS}$ and V${}_{GS}$ values results from the self-heating [34,35]. The transconductance (g${}_{m}$) is about 220 mS/mm and achieves maximum values for the expected range of operating points of transistor. The transfer characteristics show a clear pinch-off at V${}_{GS}$ = −6 V and very good linear behaviour up to V${}_{GS}$ = −2 V. The measured leakage current is about 0.1 mA/mm and can be attributed to the gate leakage current. We do not observe any additional, measurable leakage current through the buffer layers or the substrate.

**Figure 7:**
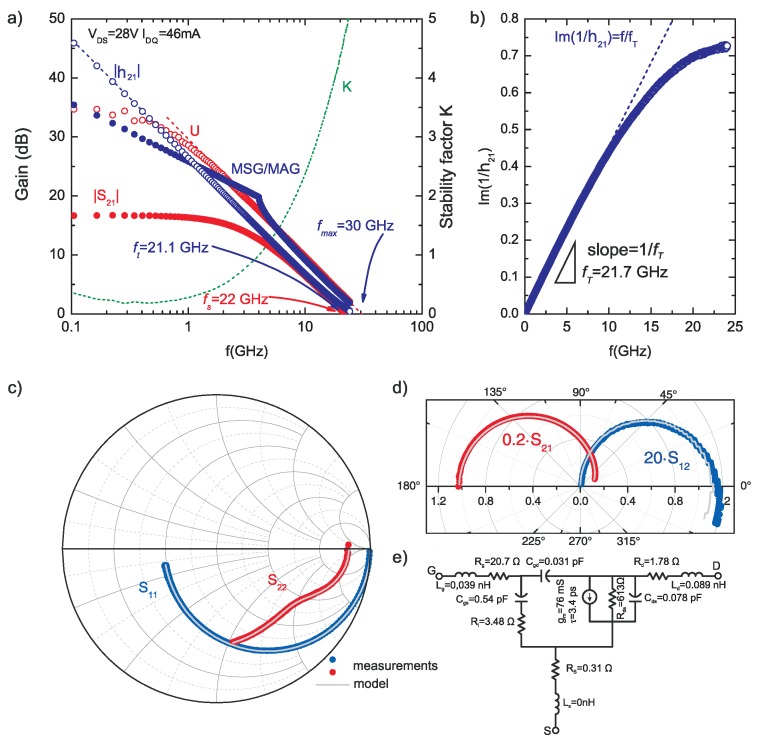
(**a**) high frequency characteristics of|S${}_{21}$|, |h${}_{21}$|, U and MSG/MAG of the fabricated AlGaN/GaN HEMT on SI Ammono-GaN substrates with Ti/Al/Mo/Au ohmic contacts with subcontact n${}^{+}$-In${}_{x}$Ga${}_{1-x}$N:Si regrown epilayer, annealed at 850 °C; (**b**) f${}_{T}$ determination using the Gummel method; (**c**) the Smith chart of $S_{11}$ and S${}_{22}$ reflection coefficients; (**d**) section of polar plot of $S_{21}$ and S${}_{12}$ transmission coefficients; (**e**) equivalent circuit with extracted parameters used for simulations.

The frequency performance of transistors was also investigated. The *S*-parameters of fabricated transistors were measured over a 45 MHz to 24 GHz frequency range using on-wafer measurement station Cascade M150 with an Agilent N5242A network analyser (Keysight, Santa Rosa, CA, USA) and 50 Ω input and output impedance. Figure 7a shows RF characteristics such as current gain (|h${}_{21}$|), maximum stable/available gain (MSG/MAG), unilateral gain (U) and |S${}_{21}$| gain at quiescent point V${}_{DS}$ = 28 V and I${}_{DQ}$ = 46 mA (115 mA/mm). The chosen operating point corresponds with condition to achieve maximum gain, at typical supply voltage used in standard GaN HEMT microwave circuits (V${}_{DS}$ = 28 V). The maximum frequency ($f_{MAX}$) and cut-off frequency ($f_{T}$) was 30 GHz and 21.1 GHz as obtained by linear extrapolation with −20 dB/dec slope of U (or MSG/MAG) and |h${}_{21}$|, respectively. The $f_{T}$ value (21.7 GHz) was also estimated using the Gummel method [36] as shown in Figure 7b. An $f_{T}$·L${}_{g}$ product of 16.8 GHz·μm was achieved. The |S${}_{21}$| gain attains 0 dB for frequency ($f_{s}$) of 22 GHz. The MAG and |S${}_{21}$| was 22.7 dB and 15.3 dB at 2 GHz and 19.8 dB and 12.7 dB at 4 GHz. It is worth noting that |S${}_{21}$| depends on the source and load impedances and the 50 Ω impedance of the measurement system is not optimal neither for maximum gain nor for maximal output power. Therefore, |S${}_{21}$| should not be used for direct comparison of the transistor structures. In order to estimate microwave properties and usability of the transistor, a small-signal model was extracted on the basis of the measured S-parameters. The measured and simulated input (S${}_{11}$) and output (S${}_{22}$) reflection coefficients are shown in Smith chart (Figure 7c) and forward (S${}_{21}$) and reverse (S${}_{12}$) transmission coefficients are plotted on the polar chart (Figure 7d). An equivalent circuit along with extracted model parameters are presented in Figure 7e. The microwave measurements indicate the lack of significant parasitic elements and confirm the high quality of fabricated HEMTs with ohmic contacts with subcontact n${}^{+}$-In${}_{x}$Ga${}_{1-x}$N:Si regrown epilayer. The g${}_{m}$ value obtained from equivalent circuit was about 80.8 mS (200 mS/mm). As the slope of the g${}_{m}$(V${}_{GS}$) curve is steep, the DC g${}_{m}$ value of 200 mS/mm corresponds to I${}_{DS}$ value of about 120 mA/mm as can be deduced from Figure 6b. This value is close to the used I${}_{DQ}$ value, and confirms correspondence between the DC characteristics and the AC model.

In order to fully compare the Ti/Al/Mo/Au and Ti/RuSi/Au ohmic contacts with subcontact n${}^{+}$-In${}_{x}$Ga${}_{1-x}$N:Si regrown epilayer, with reduced annealing temperature, another set of devices with each metallization were fabricated (on the parts of the same wafer), in a similar manner and with the same dimensions as described in the experimental section.

**Figure 8:**
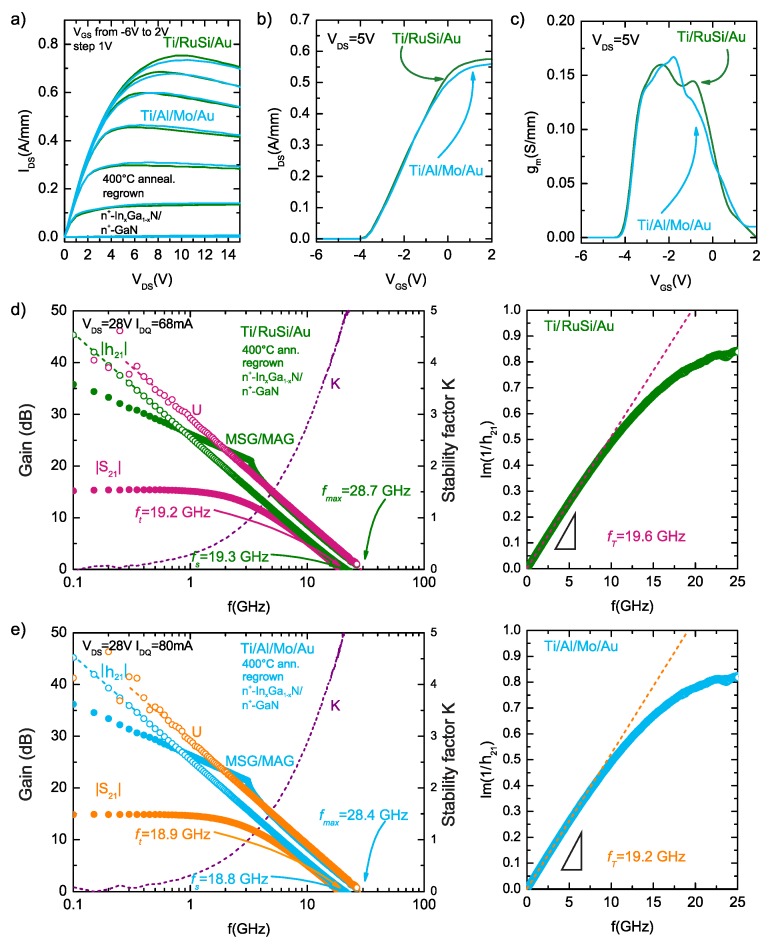
Comparison of (**a**) output and (**b**,**c**) transfer characteristics, (**d**,**e**) high frequency characteristics of |S${}_{21}$|, |h${}_{21}$|, U and MSG/MAG and f${}_{T}$ determination using the Gummel method for the fabricated AlGaN/GaN HEMT on SI Ammono-GaN substrates with Ti/Al/Mo/Au and Ti/RuSi/Au ohmic contacts with subcontact n${}^{+}$-In${}_{x}$Ga${}_{1-x}$N:Si regrown epilayer, annealed at 400 °C.

The comparison of output and transfer characteristics of AlGaN/GaN HEMTs with Ti/Al/Mo/Au and Ti/RuSi/Au ohmic contacts with subcontact n${}^{+}$-In${}_{x}$Ga${}_{1-x}$N:Si regrown epilayer, annealed at 400 °C, are shown in Figure 8a–c. The transistors with Ti/RuSi/Au metallizations have a maximum output current of 756 mA/mm as compared to 736 mA/mm for the devices with Ti/Al/Mo/Au metallizations annealed at 400 °C. For both of the transistors, the pinch-off voltage was about −3.6 V, as is not dependent on used ohmic contact metallization schemes. It is worth noting that maximum output current is lower than described earlier; however, it is not connected with ohmic contact resistance but with parameters of 2DEG for AlGaN/GaN HEMT heterostructures used for fabrication of transistors, as suggested by lower pinch-off voltage values in those devices as compared to reported earlier in the text. The maximum transconductance value was 159 mS/mm at V${}_{GS}$ = −2.32 V for Ti/RuSi/Au devices and 167 mS/mm at V${}_{GS}$ = −1.79 V for the devices with Ti/Al/Mo/Au metallizations annealed at 400 °C.

Figure 8d,e shows the comparison of high-frequency characteristics (measured at quiescent point V${}_{DS}$ = 28 V) of AlGaN/GaN HEMTs on semi-insulating Ammono-GaN substrates with Ti/Al/Mo/Au and Ti/RuSi/Au ohmic contacts with subcontact n${}^{+}$-In${}_{x}$Ga${}_{1-x}$N:Si regrown epilayer, annealed at 400 °C. As can be seen, the transistors with both Ti/Al/Mo/Au and Ti/RuSi/Au exhibit a similar good high frequency performance and extracted dynamic parameters are very similar. Observed differences in those parameters do not deviate from typical values of parameter scattering for used technology. The maximum frequency ($f_{MAX}$) and cut-off frequency ($f_{T}$) was 28.4 GHz and 18.8 GHz and 28.8 GHz and 19.2 GHz for transistors with Ti/Al/Mo/Au and Ti/RuSi/Au ohmic contacts with subcontact n${}^{+}$-In${}_{x}$Ga${}_{1-x}$N:Si regrown epilayer, annealed at 400 °C, respectively, showing comparable values as for transistors with Ti/Al/Mo/Au ohmic contacts with subcontact n${}^{+}$-In${}_{x}$Ga${}_{1-x}$N:Si regrown epilayer, annealed at high temperature of 850 °C.

### 3.3. Design and Fabrication of the on Microwave Power Amplifier Using AlGaN/GaN HEMTs on Semi-Insulating Ammono-GaN Substrates

To verify usability of the GaN HEMTs on Ammono GaN substrate in microwave designs, a class-AB power amplifier was designed using a small-signal approach based on a very popular Cripps method. This method enables a load impedance optimal for maximum output power level based on DC current-voltage characteristics and small-signal output impedance at a given transistor operating point to be determined [37]. The impedance condition recommended by Cripps suggests a series circuit, as the load, leaving aside the actual structure of transistor output circuit, which in the MESFET (metal semiconductor field effect transistor) and HEMT case has parallel circuit–parallel connection of C${}_{DS}$ and R${}_{DS}$ on the equivalent circuit (Figure 7e). Therefore, the definition of admittance condition in the plane of C${}_{DS}$ and R${}_{DS}$ elements seems natural. This approach is described in detail in [38,39]. In the assembly of the amplifier circuit, we use AlGaN/GaN HEMT on semi-insulating Ammono-GaN substrate with subcontact n${}^{+}$-In${}_{x}$Ga${}_{1-x}$N:Si regrown epilayer and Ti/Al/Mo/Au ohmic contact annealed at 850 °C (representative characteristics of one of the devices from the same wafer are presented in Figure 6 and Figure 7).

**Figure 9:**
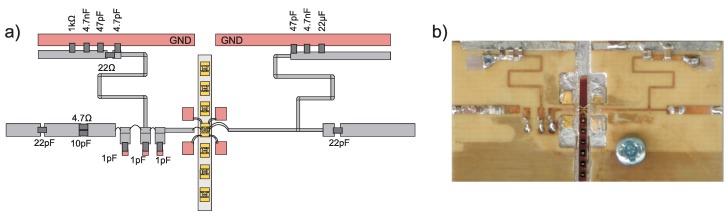
(**a**) assembly schematic and (**b**) photography of the fabricated amplifier.

According to the aforementioned procedure, the amplifier was designed in a Keysight Advanced Design System (ADS) environment in the microstrip technique on Rogers RO4003C laminate (Chandler, AZ, USA) with h = 0.02 and $\epsilon_{r}$ = 3.35. The output matching section was optimized to fulfill maximum power condition while the input matching circuit was optimized for minimum input return loss. The input network contains lossy elements to stabilize the amplifier. The assembly schematic and photography of the fabricated amplifier are presented in Figure 9, respectively. The use of a F&S BONDTEC 5632 bond machine (Braunau, Austria) ensures good control over the length and shape of bond wires. This is confirmed by the excellent compliance of simulations and measurements.

**Figure 10:**
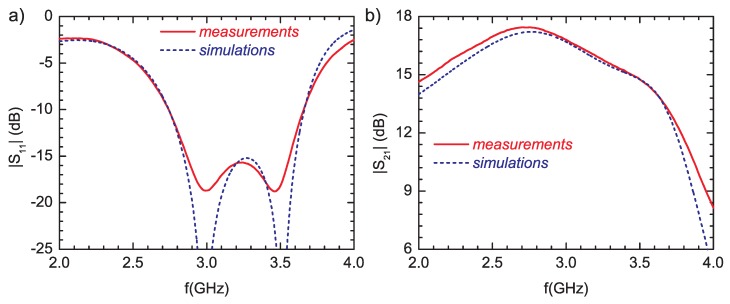
(**a**) the reflection coefficient |S${}_{11}$| and (**b**) the small-signal gain |S${}_{21}$| of the amplifier.

The small-signal measurements were performed over a 2 GHz to 4 GHz frequency range and for input power level of −10 dBm. The simulations and measured results of the amplifier are compared in Figure 10.

**Figure 11:**
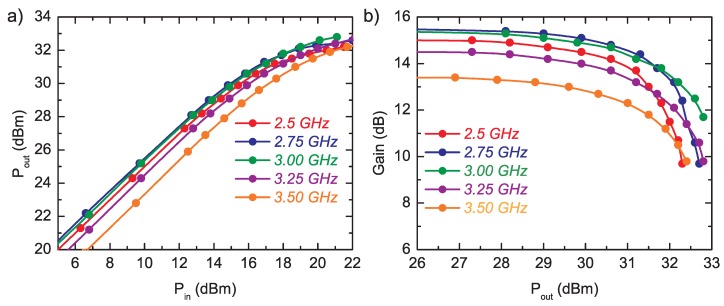
Transfer characteristics for continuous wave (CW) excitation—(**a**) output power P${}_{out}$ = fP(${}_{in}$) and (**b**) gain G = f(P${}_{OUT}$).

**Figure 12:**
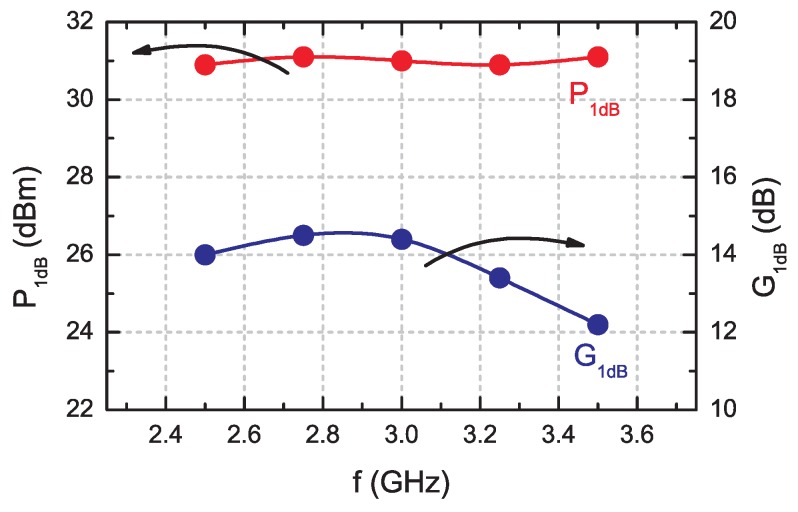
The output power at 1-dB gain compression point with associated gain of the amplifier.

The power transfer characteristics P${}_{out}$(P${}_{in}$) of the amplifier measured for continuous wave (CW) mode from 2.5 GHz to 3.5 GHz frequency range are shown in Figure 11. Despite the gain irregularity, the available power is more equal. The output power at 1 dB gain compression (1 dB G.C.P.) point with associated gain vs. frequency is shown in Figure 12. In the whole frequency range, 1 dB G.C.P. is 30.3 ± 0.1 dBm.

The maximum output power of 32.2 dBm (1.66 W) was obtained at 3 GHz for the RF ON bias point V${}_{DS}$ = 28 V and I${}_{DS}$ = 150 mA (shallow class AB) of the transistor. Due to the high GaN HEMT chip thickness of 400 μm, the thermal conditions inside the transistor result in a drain current drop. It is the main limitation to obtaining higher output power level as well as higher efficiency. To reduce thermal resistance R${}_{th}$ value, the chip thickness should be decreased e.g., to standard used thickness of 100 μm. Additionally, source to ground connection is made using bond wires that decrease gain. Going through metal via holes from source pads to ground can improve the heat dissipation and gain, and reduce R${}_{on}$.

The parameters of the amplifier show a high quality of fabricated transistors. The output power level is higher than 30 dB m at 1 dB G.C.P. over a 2.5 GHz to 3.5 GHz frequency range with 13.5 ± 1.0 dB gain. The maximum output power density of 4.15 W/mm is comparable with commercially available GaN HEMTs e.g., Wolfspeed CGH6008D 3.8 W/mm (Durham, NC, USA) [40], Qorvo TGF2023-2-01 4.8 W/mm) (Greensboro, NC, USA) [41].

## 4. Conclusions

In this work, AlGaN/GaN HEMTs, with active layers homoepitaxially grown on semi-insulating Ammono-GaN substrates, were fabricated. The use of regrown, highly-doped In${}_{x}$Ga${}_{1-x}$N/GaN sub-contact layers resulted in decreasing the contact resistivity from 0.8–1.1 Ω·mm (as for recessed ohmic contacts) to 0.3–0.6 Ω·mm and decreasing value of parasitic elements like source and drain resistance (see Table 1). This leads to enhancement of DC and RF performance as compared to AlGaN/GaN HEMTs on semi-insulating Ammono-GaN substrates with recessed Ti/Al/Mo/Au ohmic contacts [23]. An 1 A/mm on-state current was achieved and $f_{T}$ and $f_{MAX}$ were 21 and 30 GHz, respectively, for 0.8 μm gate length devices. Moreover, thanks to the use of selectively regrown highly-doped In${}_{x}$Ga${}_{1-x}$N/GaN:Si layers, it is possible to use new types of metallization, e.g., with potentially increased thermal stability like Ru-based metallization schemes or to reduce the thermal budget to obtain low resistivity ohmic contacts, which is not possible to achieve using conventional technologies. Those ohmic contacts exhibit very good electrical parameters (R${}_{c}$ = 0.38–0.43 Ω·mm) and allow for obtaining transistors with DC and high-frequency parameters comparable to those for devices with Ti/Al/Mo/Au ohmic contacts with subcontact n${}^{+}$-In${}_{x}$Ga${}_{1-x}$N:Si regrown epilayer, annealed at high temperature of 850 °C.

Overall, we have shown AlGaN/GaN high electron mobility transistors fabricated on truly bulk monocrystalline semi-insulating GaN substrates with parameters (output current and output power density) comparing well with devices manufactured on silicon carbide substrates available from commercial manufacturers. The high potential of developed technology was proved by assembly of microwave systems using our devices (S-band power amplifiers). The fabricated S-band power amplifiers with AlGaN/GaN HEMTs on semi-insulating Ammono-GaN substrates have maximum power density of 4.15 W/mm. The high power and high frequency performance can be further enhanced by optimizing gate design and length and formation of via hole interconnections. 

## Figures and Tables

**Table 1:** Comparison of ohmic contact resistances (R${}_{C}$), and source and drain resistances (R${}_{S}$ and R${}_{D}$) obtained from S-parameters of AlGaN/GaN high electron mobility transistor (HEMT) on SI Ammono-GaN substrates with Ti/Al/Mo/Au ohmic contacts with subcontact n${}^{+}$-In${}_{x}$Ga${}_{1-x}$N:Si regrown epilayer and with recessed Ti/Al/Mo/Au ohmic contact.

Ohmic Contact	R${}_{C}$ (Ω·mm)	R${}_{S}$ (Ω)	R${}_{D}$ (Ω)
recessed Ti/Al/Mo/Au	0.8–1.1	0.86	3.4
Ti/Al/Mo/Au with regrown epilayer	0.3–0.6	0.31	1.78
